# Redox Regulation and Oxidative Stress: The Particular Case of the Stallion Spermatozoa

**DOI:** 10.3390/antiox8110567

**Published:** 2019-11-19

**Authors:** Fernando J. Peña, Cristian O’Flaherty, José M. Ortiz Rodríguez, Francisco E. Martín Cano, Gemma L. Gaitskell-Phillips, María C. Gil, Cristina Ortega Ferrusola

**Affiliations:** 1 Laboratory of Equine Reproduction and Equine Spermatology, Veterinary Teaching Hospital, University of Extremadura, 10003 Cáceres, Spain; jmortizro@gmail.com (J.M.O.R.); femartincano@gmail.com (F.E.M.C.); gemmagaitskell@hotmail.com (G.L.G.-P.); crgil@unex.es (M.C.G.); cristinaof@unex.es (C.O.F.); 2 Departments of Surgery (Urology Division) and Pharmacology and Therapeutics, Faculty of Medicine, McGill University, Montréal, QC H4A 3J1, Canada; cristian.oflaherty@mcgill.ca

**Keywords:** horses, spermatozoa, reactive oxygen species (ROS), oxidative stress, redox regulation, equine

## Abstract

Redox regulation and oxidative stress have become areas of major interest in spermatology. Alteration of redox homeostasis is recognized as a significant cause of male factor infertility and is behind the damage that spermatozoa experience after freezing and thawing or conservation in a liquid state. While for a long time, oxidative stress was just considered an overproduction of reactive oxygen species, nowadays it is considered as a consequence of redox deregulation. Many essential aspects of spermatozoa functionality are redox regulated, with reversible oxidation of thiols in cysteine residues of key proteins acting as an “on–off” switch controlling sperm function. However, if deregulation occurs, these residues may experience irreversible oxidation and oxidative stress, leading to malfunction and ultimately death of the spermatozoa. Stallion spermatozoa are “professional producers” of reactive oxygen species due to their intense mitochondrial activity, and thus sophisticated systems to control redox homeostasis are also characteristic of the spermatozoa in the horse. As a result, and combined with the fact that embryos can easily be collected in this species, horses are a good model for the study of redox biology in the spermatozoa and its impact on the embryo.

## 1. Introduction

The male gamete, the spermatozoon, is generated in the germinal epithelium of the testes in a process called spermatogenesis. This epithelium consists of germ cells in different stages of development, intermingled with Sertoli cells that provide structural support and nursing, protecting the germ cells. Spermatogenesis is initiated by the differentiation of spermatogonia from a stem cell pool. These cells initiate a proliferative phase entering a continuous process of mitotic division, dramatically increasing spermatogonial numbers. This process is usually termed spermatocytogenesis. In the next step, cells enter a meiotic phase that includes duplication and exchange of genetic information and two meiotic divisions which reduce the chromosome complement to form round haploid spermatids. During the spermiogenesis phase, round spermatids experience a dramatic transformation that includes compaction and silencing of DNA and elongation of the nucleus, development of specific structures such as the sperm tail and acrosome, relocation of the mitochondria in the midpiece, in addition to the loss of other organelles and most of the cytoplasm. Fully developed spermatozoa are released in the lumen of the seminiferous tubules in a process termed spermiation. Recent reviews on this topic can be found elsewhere [1,2,3,4]. Chemically, oxidation is the loss of an electron, while reduction is the gain of an electron. This nomenclature reflects the tendency of oxygen, a highly electronegative atom, to partially or fully steal an electron from other molecules. Reactive oxygen species (ROS) [5,6] are atoms or molecules with a single unpaired electron, including, among others, superoxide (O_2_•^−^), the hydroxyl radical (HO•) and the lipid peroxide radical (LOO•). Although hydrogen peroxide (H_2_O_2_) is not a free radical, it is a precursor of HO•. UV radiation and the presence of metal ions (Fe^2+^, Fe^3+^ or Cu^2+^) generate HO•. All aerobic organisms depend on the generation of ATP from electrochemical energy generated in the four electron reduction of molecular oxygen into water. During this process the mitochondrial transport chain may lose electrons, leading to the formation of ROS.

Moreover, mitochondrial dysfunction may exacerbate the loss of electrons and thus increase the production of ROS to toxic levels disrupting redox homeostasis [6]. This particular effect is especially critical in horses. The stallion spermatozoon is characterized by an unusually intense mitochondrial activity in comparison with other mammals [7,8,9,10,11]. 

Spermatozoa were the first cells known to be capable of generating ROS [12]. This early report demonstrated that bovine spermatozoa produce H_2_O_2_ as a consequence of cellular respiration. It also showed that the production of H_2_O_2_ inhibits respiration and concluded that bovine spermatozoa must be equipped with a mechanism for the elimination of H_2_O_2_ at a low rate, to keep it at physiological levels. For a long time, the production of ROS was considered solely as a toxic byproduct of sperm metabolism; however, nowadays, extensive evidence indicates that crucial functions of the spermatozoa are redox regulated, and redox regulation has become a major area of research in sperm biology [13,14,15,16,17,18,19,20]. Since the discovery of ROS production by the spermatozoa, the concept of oxidative stress has evolved, and enormous research interest in this topic has developed in the last decade. As an example, a recent search in PubMed retrieved 215842 entries using the term oxidative stress, when this term was combined with spermatozoa 2777 entries were obtained (https://www.ncbi.nlm.nih.gov/pubmed/, accessed September, 1 2019). Under aerobic conditions, production of ROS is unavoidable. However, organisms have evolved to develop complex mechanisms to maintain the production of ROS at physiological levels (oxidative eustress) and the redox signaling dependent on ROS regulated [21,22,23]. Interestingly, the ability to respond to ROS appeared very early in the course of evolution, well before the increase of atmospheric oxygen, probably in response to low ozone levels, since U.V. radiation splits water into ROS [24]. 

## 2. Sources of ROS in the Spermatozoa

Several pathways lead to the generation of ROS, including the production of O_2_^−^•, H_2_O_2_, reactive nitrogen species (RNS), and OH• [25]. The superoxide anion is generated from the coupling of O_2_ with an electron (e^−^). The electron donor is usually NADH or NADPH, and the reaction is catalyzed by various oxidases; NADPH oxidases, xanthine oxidase and complex I/II/III/IV from the mitochondria [25]. The generation of H_2_O_2_ occurs after the dismutation of O_2_^−^•, mostly catalyzed by superoxide dismutases (SODs), although a small percentage occurs spontaneously. Some oxidases also have dismutase activity and may contribute to direct production of peroxide from superoxide. The reaction of O_2_^−^• with reduced transition metals may lead to formation of H_2_O_2_ [25]. Most of the OH• is generated from H_2_O_2_ and O_2_^−^• in a reaction catalyzed by a metal ion (iron or cupper). This is known as the Habor–Weiss reaction. This reaction occurs in two steps; in the first step, O_2_^−^• reduces Fe^3+^ to Fe^2+^ (Fe^3+^ O_2_^−^•→ Fe^2+^ + O_2_), and the second step is the Fenton reaction where Fe^2+^ reacts with H_2_O_2_ to generate OH• and OH^-^ (Fe^2+^ + H_2_O_2_ → Fe^3+^ + OH• + OH^−^) [25]. Nitric oxide and ONOO^−^ (form by the combination of NO and O_2_^−^•) are the most important RNS in spermatozoa [25].

Several potential sources can be responsible for ROS production in the spermatozoa, including the spermatozoa itself and contaminating cells in the ejaculate. Dead spermatozoa are a major source of ROS, frequently overlooked in reproductive technologies [26]. l-amino oxidase (LAAO) is present in stallion spermatozoa being able to generate significant amounts of ROS; aromatic amino acids are substrates for this enzyme, producing substantial amounts of ROS, especially in the presence of dead spermatozoa [26]. Interestingly, cryopreservation media contain sufficient amounts of aromatic amino acids to activate this enzyme. Ongoing proteomic studies in our laboratory have also confirmed the presence of this enzyme in stallion spermatozoa. A NADP oxidoreductase system has been detected in the membrane [27], however nowadays it is considered that the main source of reactive oxygen species is electron leakage in the mitochondrial electron transport chain (ETC) [7,8,10,28,29,30,31]. In particular, defective mitochondria may represent a hallmark of male infertility. Evidences of mitophagy in human sperm were described in our laboratory, suggesting that activation of mitophagy is a mechanism that maintains proper sperm function [32]. The sources of reactive oxygen species in the electron transport chain of the stallion spermatozoa have also recently been investigated in our laboratory [9,10], confirming the role of the ETC as a main source of ROS in stallion spermatozoa. 

## 3. Redox Regulation and Signaling 

Although initially, oxidative stress was defined as a disturbance in the pro-oxidant-antioxidant balance in favor of the former, current knowledge has evolved and oxidative stress is better defined in terms of regulation of redox signaling. Numerous processes are redox regulated in biological systems. Redox regulation is similar to pH regulation, the pH varies in different cellular compartments, also the redox state is not an overall redox state and vary in different compartments of the spermatozoa [33]. Redox reactions consist of the transfer of electrons (e^−^) from one molecule (oxidation) to another molecule (reduction). Thus, reduction implies a decrease in overall charge (more e^−^) of the molecule, while oxidation implies an increase in overall charge (fewer e^−^). Reactive oxygen species, such as the superoxide anion O_2_^−^•, are low molecular weight compounds that are chemically unstable, particularly in biological systems [21]. The hydroxyl radical is the most reactive and oxidizes virtually any closer molecule. The reactivity of HO• is 7 × 10^9^ L mol^−1^ s ^−1^, while the rate constant for O_2_^−^• is <0.3 and is 2 × 10^−2^ L mol^−1^ s^−1^ for H_2_O_2_ [33]. Another electronically excited state of interest in spermatology is singlet molecular oxygen, generated by photoexcitation mainly by ultraviolet A and B light rays, but even infrared and visible light may also generate photobiological responses. This is the rationale of the customary procedure of avoiding light exposure during semen processing [33]. Other species include alkoxyl and peroxyl radicals, non-radical species such as hypochlorite, peroxynitrite, singlet oxygen and lipid peroxydes, among others [34]. To understand the basis of redox signaling it is important to bear in mind the characteristics of different ROS. As previously mentioned the HO• is the most reactive, and has the shortest half-life (10^−15^ s.) [24]. The HO•, is considered to be the most harmful oxidant, with no signaling functions. Although O_2_^−^• may have difficulty diffusing through membranes due to its anionic charge, it may use specific channels in some tissues [35,36,37]. Hydrogen peroxide is a stable compound and in addition is a nonpolar molecule that can easily diffuse through membranes, and is also transported through aquaporin channels [24,38,39,40]; all of which make H_2_O_2_ a suitable molecule for redox signaling. The primary target of hydrogen peroxide is the thiol group of the amino acid cysteine, which is oxidized in a reversible fashion. The presence of glutathione (GSH) and other thiols in spermatozoa is well known [41], also the role of oxidative regulation in significant biological processes occurs in very early stages of development. For example, studies in sea urchin, show an oxidative burst that occurs at the time of fertilization preventing polyspermy through the activation of a dual oxidase (Udx1), that induces cross linking of surface proteins on the egg surface [42,43]. Also, oxidation reduction processes of sulfhydryl groups of protamines are critical for chromatin condensation during spermatogenesis [44]. 

Nitric oxide is a ubiquitous free radical generated from the oxidation of l-arginine to l-citruline by three isoforms of reduced nicotinamide adenine dinucleotide phosphate (NADPH)-dependent NO-synthases (NOS) [45]. Among other functions, NO is relevant for spermatogenesis, penile erection, folliculogenesis, and ovulation [46]. In spermatozoa, NO appeared to play a major role in the regulation of sperm motility and capacitation [47,48,49]. Studies in our laboratory have identified the presence of NOS in stallion spermatozoa, its role in sperm functionality and, interestingly, we also showed the effect of egg yolk present in freezing extenders scavenging NO [50]. While the NO produced by NOS is a messenger molecule, it may react with O_2_^−^• to form peroxynitrite (ONOO^−^) [33], an oxidant that may induce 3-nitrotyrosine residues in proteins, affecting mitochondrial functions and triggering cell death via oxidation and nitration reactions [51]; however, due to the high content of SOD (1000 times more than intracellular NO levels), the production of ONOOO^−^ is prevented by the rapid dismutation of O_2_^−^• [25].

Many cellular processes are redox regulated. In spermatozoa, redox regulation has been extensively studied in relation to capacitation [13,15,52,53,54,55,56,57]. Capacitation is the maturational process that sensitizes spermatozoa to recognize and fertilize the oocyte. Capacitation involves, removal of cholesterol from the plasma membrane, removal of coating materials from the membrane, a rise in intracellular Ca^2+^, an increase in intracellular cAMP, and a dramatic increase in tyrosine phosphorylation.

Removal of cholesterol from the membrane is preceded by its oxidation, stimulated by bicarbonate, and the formation oxysterols [58,59,60] that are depleted from the sperm membrane by albumin. Different aspects are worth mentioning in the context of the present review; one is the fact that bovine studies have demonstrated that after freezing and thawing this oxidative mechanism is altered, offering an explanation of the reduced fertility of cryopreserved spermatozoa [61]. The stallion spermatozoa present difficulties to capacitate in vitro, explaining the poor results of conventional IVF in this species. This issue has been the subject of an excellent recent review [62], and the reader is referred to it for detailed information in the topic; however, the possibility that this may relate to the specific redox regulation in spermatozoa is an intriguing possibility that warrants to be further explored; interestingly, intracellular glutathione (GSH) is much higher in horses than in other domestic species. Also, during capacitation the sperm plasma membrane potential (E(m)) hyperpolarizes [56,63,64], and spermatozoa experience alkalinization. Detailed reviews on the molecular aspects of capacitation can be found elsewhere [17]. Interestingly, only a subpopulation of spermatozoa is able to experience capacitation [52,56]. Tyrosine phosphorylation is a redox regulated process [17,20,54,65,66,67,68,69,70]. Other functions of the spermatozoa, such as activated motility may also be redox regulated [17,71], in relation to tyrosine phosphatases (PTPs), which are intracellular targets for ROS [72]. The activity of PTPs depends on a conserved cysteine (Cys) residue, where oxidation results in the inactivation of the enzyme [22,73]. On the other hand, ROS can also activate kinases. In addition to hydrogen peroxide, other species such as reduced glutathione (GSSG), hydrogen sulphide and lipid peroxides (LPO) can inactivate PTPs [74]. Reversible oxidation of target cysteine residues in specific proteins modulates its activity [22]. In order to function in a reversible manner oxidized cysteine (Cyss) residues need to be reduced. This reversibility depends on adequate availability of reducing molecules including the peroxiredoxin (PRDX) family of antioxidant enzymes [22]. Peroxirredoxins have been described in spermatozoa [13,14,15,75] and play a major role in sperm function, stressing the importance of redox signaling in these highly specialized cells. Reversing the oxidized Cys residue in this family of pathways involves thiorredoxin or GSH. Reduction of the higher oxidation state (sulphinic acid SO_2_H) may require sulfiredoxin or sestrins [22,76]. This reversible sequential oxidation of PRDXs allows a tight regulation of the function of these proteins in a regulation described as a “floodgate” model [77,78]. Spermatozoa are rich in thiols [41], with the majority of thiol groups associated with proteins, which may suggest that redox regulation is an important regulatory mechanism in these cells. Spermatozoa are transcriptionally silent cells whose regulation depends on post transcriptional modification of proteins. One interesting example, since mitophagy has been recently described in spermatozoa [32], of proteins regulated by reversible oxidation of Cys residues, is the large family of Cys-dependent proteases [22]. In particular, the cysteine protease HsAtg4 is a direct target for oxidation by H_2_O_2_, specifically a residue located near the protein’s catalytic site [79]. The presence of a similar mechanism in spermatozoa is an intriguing possibility and deserves further research [32]. Other functions in the spermatozoa that are redox regulated, include control of motility [71], and binding to the oviductal epithelium to form the sperm reservoir [80,81,82].

## 4. Modern Concept of Oxidative Stress Applied to Spermatozoa 

Since redox regulation is being unveiled as a major mechanism regulating sperm function, probably at the same level as tyrosine phosphorylation and other post translational modifications of sperm proteins, sophisticated mechanisms must be present to maintain redox status under physiological control. Both seminal plasma and the spermatozoa itself contain enzymatic and non-enzymatic systems that contribute to maintenance of oxidative eustress. Recent research from our laboratory shows that in stallion spermatozoa seminal plasma plays a major role in regulating redox status. The steady state redox potential (E_h_) can be estimated using the Nerst Equation: Eh = Eo+ RT/Ln [oxidized molecule/reduced molecule], where Eo is the standard reduction potential, R = gas constant, T is the absolute temperature, *n* = number of electrons transferred and F is the Faraday constant [23]. Recently, a system to easily measure the steady state in semen has become available and is being introduced into reproductive medicine and clinics. Using this system, E_h_ is provided as the static oxidation reduction potential (sORP) and is expressed as millivolts per million spermatozoa. E_h_ in raw semen (seminal plasma present) was measured and was found to be 1.62 ± 0.06 mV/10^6^ spermatozoa, when seminal plasma was removed, it was 7.9 ± 0.79 mV/10^6^ spermatozoa, thus showing a much higher overall oxidation status [83]. This finding suggests that regulation of the extracellular medium may also be of great importance as is the case in other cells [83], from this viewpoint it is well recognized that equine seminal plasma is rich in antioxidants [84,85,86,87,88,89]. On the other hand, it is important to consider that once the semen is deposited in the mare’s uterus or is processed, the antioxidants in seminal plasma are removed from close contact with the spermatozoa, meaning the importance of intrinsic antioxidant defenses in the spermatozoa become critical [13,15,90,91,92]. 

The spermatozoa itself also has antioxidant defenses, including glutathione, and other enzymatic antioxidant defenses such as the paraoxonase [93,94,95,96,97], thioredoxin [15,98,99,100,101,102,103,104] and peroxiredoxin [13,14,51,75,90,91,105,106] families of proteins. Ongoing proteomic studies in our laboratory have identified peroxiredoxins 5 and 6, and thioredoxin reductase in stallion spermatozoa. Interestingly, and as previously indicated, the concentration of intracellular GSH in the horse spermatozoa is higher than in most domestic species. A recent study in our laboratory revealed that the mean concentration of GSH in stallions was 8.2 ± 2.1 μM/10^9^ spermatozoa [107], while values reported in other species are in the nanomolar ranges per billion spermatozoa [41]. These high levels of GSH in stallion spermatozoa, may be linked to the intense mitochondrial activity of the spermatozoa in this species. Intense mitochondrial activity causes increased ROS production, and thus sophisticated mechanisms to maintain redox homeostasis may have evolved differently between species with spermatozoa less dependent on oxidative phosphorylation for ATP production. In relation to this, evidence of the presence and activity of the Cystine antiporter SLC7A11 in stallion spermatozoa has been discovered [83]. This antiporter exchanges extracellular cystine (oxidized form of cysteine) for intracellular glutamate. Once in the cell, cystine is reduced and used for GSH synthesis. Indirect evidence of the presence of a system exporting glutamate in spermatozoa were reported as early as in 1959 [108]. Evidence of GSH synthesis in stallion spermatozoa [107], include the presence of the enzymes glutathione synthetase (GSS) and gamma glutamylcysteine synthetase (GCLC). In addition, functional studies indicate their activity; the use of the specific inhibitor l-Buthioninine sulfoximide (BSO) reduced GSH synthesis from cysteine. In this particular experiment, mass spectrometry (MS) was used to specifically identify GSH and avoid interference with other thiols. Overall these results point to a sophisticated redox regulation in stallion spermatozoa. It is considered that most extracellular cysteine is present in the disulfide form (cystine), thus the presence of the xCT/SLCTA11 antiporter may be a major mechanism of cystine incorporation in the spermatozoa. This antiporter is present and active in stallion spermatozoa [83]. In addition to its role in the incorporation of cysteine for GSH synthesis, a potential role in an active Cys/Cyss redox node in the spermatozoa must be considered. Overall, these recent findings support the hypothesis of a complex redox regulation in the spermatozoa. Oxidative stress is thus better defined as the fail in the regulation of redox signaling due either to overproduction of ROS, or exhaustion of regulatory mechanisms. This latter point has recently been addressed, and functionality of the stallion spermatozoa is linked to thiol content. When thiols are exhausted stallion spermatozoa rapidly enters senescence, which is characterized by increased production of lipid peroxides, activation of caspase 3, loss of motility and death [109,110]. Remarkably, this senescence is triggered by ROS as is capacitation. It has been proposed that both processes are linked. Only one capacitated spermatozoa will fertilize the oocyte, while the redundant spermatozoa finally succumb in a truncated apoptotic cascade, characterized by enhanced mitochondrial ROS production, lipid peroxidation, caspase activation, loss of motility and phosphatidylserine externalization, representing a signal to phagocytic cells for the elimination of redundant spermatozoa without significant inflammatory reaction [111].

The stallion spermatozoa is a paradigm of this sophisticated redox regulation; recent research has shown apparently paradoxical results, in this regard more fertile spermatozoa show increased ROS production [8], further underlining the concept that a tightly controlled redox regulation occurs in stallion spermatozoa. 

## 5. The Mitochondria in Redox Signaling

Electrons can be prematurely leaked to oxygen in the ETC or associated to catabolism of substrates [112,113]. Depending of the number of electrons being leaked, different outcomes are possible. If leaked one by one they generate superoxide radicals, if in pairs they generate hydrogen peroxide. When they are properly transferred four at a time, they generate water and drive OXPHOS at complex IV of the ETC. A growing body of scientific evidence is stressing the role of proper mitochondrial function in sperm physiology [7,9,10,11,28,31,32,114,115,116,117,118]; moreover, definition of oxidative stress as the result of mitochondrial malfunction, states that it is the result of “a dysfunction of electron transfer reactions leading to oxidant/antioxidant imbalance and oxidative damage to macromolecules” [119]. This theory states that O_2_^−^• does not accidentally leak from the ECT, but instead is a signaling molecule [119]. Recent research in our laboratory with an aryl hydrocarbon receptor deficient (AhR^−/−^) mouse strain, showing males of unusually high fertility (also in terms of number of pups born) revealed that this strain was characterized by higher mitochondrial activity [120]. Other reports also link mitochondrial activity with fertility in humans and equines [7,8,28,31,116,121,122]. Interestingly, the mitochondria are the more sensitive structure in the spermatozoa to stress induced by different biotechnologies, and have been proposed as a sensitive marker of sperm quality and fertilization ability [120]. Mitochondrial roles in the spermatozoa may include Ca_2_^+^ storage and signaling, production of ATP, control of sperm lifespan and activation of a specific form of apoptosis for silent, non-inflammatory elimination of redundant spermatozoa after insemination, and potentially control of redox signaling. Numerous evidences point to mitochondria as the hallmark of fertile spermatozoa. However, proper evaluation of mitochondrial function in spermatozoa is still elusive, and rarely performed under clinical settings. Fluorescent probes and flow cytometry represent the method of choice to study mitochondrial function in spermatozoa, with the potential for analysis of thousands of spermatozoa and simultaneous functions in every single spermatozoon, together with the recent development of computational methods [29] to study sperm subpopulations makes this technique the gold standard. However, technical difficulties preclude its wider use in reproductive medicine. These difficulties relate to special characteristics of commonly used probes, such as the JC-1. This dye is difficult to compensate using the 488 nm excitation laser due to the spectral characteristics of the fluorochrome, and the dual excitation depending on the formation of monomers (low mitochondrial membrane potential) of aggregates (high mitochondrial membrane potential). This particular issue can be addressed using dual excitation; monomers with the blue 488 nm laser, and aggregates with the 561 nm yellow laser. The application of computational methods to the analysis of data also improves the identification of specific spermatic subpopulations. The production of hydrogen peroxide in stallion mitochondria have been investigated in our laboratory [10], inhibition of complex I of the ETC increased the production of mitochondrial superoxide and hydrogen peroxide, suggesting that mitochondrial malfunction is a potential source of redox deregulation in stallion spermatozoa. The inhibition of complex III also caused increased ROS production. In addition, the above-mentioned study underpinned the importance of cautious selection of probes to assess ROS in spermatozoa. However, mitochondrial dysfunction may lead to either reduced or increased production of ROS [112] depending on the cause of the dysfunction and caution interpreting the results of the analysis of ROS production in spermatozoa is always advised. Specific antioxidant defenses in the mitochondria of the stallion spermatozoa include mitochondrial GSH, peroxiredoxin 5 and manganese-dependent superoxide dismutase (Mn-SOD). Mitochondrial ROS have been implicated in numerous signaling pathways in somatic cells [112] and is also likely that these species may participate in signaling in spermatozoa. Together with its importance in sperm regulation, the special characteristics of the spermatozoa, a cell devoid of most organelles and a very limited cytoplasm, may also mean this cell is a suitable model for the study of mitochondrial function. 

## 6. Redox Regulation and Sperm Metabolism

Together with mitochondria, in recent years stallion sperm metabolism have been of increased interest for scientists focused in equine reproduction [11,117]. Mitochondria play major roles in cellular metabolism, being the energetic power-house of the cell [123]. Oxidative phosphorylation (OXPHOS) and the tricarboxylic acid cycle (TCA cycle) are well known mitochondrial functions. Recent specific research in horses has underlined the importance of mitochondria as a provider of energy in the form of ATP, and the consequences it has for sperm physiology and the functional evaluation of the spermatozoa [10]. Early studies suggested that spermatozoa were glycolytic cells, however the participation of oxidative phosphorylation in production of energy is now acknowledged [9]. Early studies also suggested that ATPs produced by mitochondrial respiration could not reach distal parts of the flagellum. To solve this problem, shuttle systems and/or glycolysis ought to be present [124]. Also, species specific strategies occur in the predominance of one energy source. Recent proteomic studies indicate that the spermatozoa can use different substrates for energy, possessing the ability to oxidize fatty acids [125,126]. The stallion spermatozoa is considered to predominantly use OXPHOS for the generation of energy [7,8,11,117]. The adenine nucleotide translocator (ANT) catalyzes the transmembrane exchange of ATP, generated by oxidative phosphorylation, for cytosolic ADP [127]. Inhibition of this protein leads to reduced sperm motility suggesting that ATP produced by OXPHOS in the mitochondria plays an important role in spermatic motility in horses. Further studies aimed to clarify the role of mitochondrial ATP in stallion sperm motility. Inhibition of OXPHOS reduced spermatic motility and ATP content in stallion but not in human spermatozoa suggesting species specific differences in energetic metabolism [8]. Moreover, this study showed paradoxical relations between fertility and oxidative stress, fertile stallions were characterized by spermatozoa showing increased levels of 8-hydroxiguanidine and O_2_^−^•. These increased levels were attributed to increased mitochondrial activity in the spermatozoa of fertile stallions [8]. The relation between increased mitochondrial activity and ROS production has also been confirmed in independent studies [11]. In addition, and in line with these findings, a dramatic decrease in sperm ATP content after mitochondrial uncoupling and inhibition of mitochondrial respiration was reported [9]. Reduction of ATP was accompanied by low motilities and velocities, and interestingly, inhibition of mitochondrial respiration at the ATP synthase complex collapsed sperm membranes. This may relate to the high ATP consumption necessary to maintain the activity of the Na^+^-K^+^ ATPase pump in the spermatozoa [128]. The relation between ROS production and mitochondrial activity was also confirmed. Despite the predominance of OXPHOS, glycolysis and other sources of energy are also present in the spermatozoa. OXPHOS takes place in the mitochondria located in the sperm midpiece, while glycolysis occurs mainly in the flagellum in which the fibrous sheath is rich in glycolytic enzymes where they are anchored [129,130,131]. The substrate for glycolysis is glucose, which is incorporated into the spermatozoa through diverse glucose transporters (GLUTs) [132]. Oxidative phosphorylation uses diverse sources of substrates derived from the metabolism of carbohydrates, lipids and amino acids. While for a long time a debate has existed among spermatologists regarding the main source of energy in spermatozoa, the existence of different bioenergetic strategies in different species is now becoming clear [133], and thanks to the introduction of the “omics” technologies into spermatology, the spermatozoa is being unveiled as a cell with a much higher bioenergetic plasticity that previously assumed [126,134]. In this regard, recent proteomic studies in horses and humans reveal that beta oxidation of fatty acids plays an important role in providing energy for the spermatozoa [126,135]. The pentose phosphate cycle pathway (PPP) is also present in spermatozoa [133,136,137,138,139,140,141]. NADPH produced by the PPP is important for the re-activation of 2-CysPRDXS. [90] In human spermatozoa, the pentose phosphate pathway can respond dynamically to oxidative stress [142] and the inhibition of glutathione reductase impairs the ability of sperm to resist oxidative stress and lipid peroxidation [140]. Also, NADPH may play a role in relation to the activity of an NADPH oxidase which plays a role in capacitation [137]. The glutathione peroxidase-glutathione reductase-pentose phosphate pathway system is functional and provides an effective antioxidant defense in normal human spermatozoa [140,143]. Overall, current knowledge on sperm metabolism suggests species specific differences and a great metabolic plasticity in the spermatozoa, which are able to adapt their metabolism to the changing environments that they are exposed to, on their travel to fertilize the oocyte. Recent research using the strategy of intervention on the metabolic flexibility of stallion spermatozoa seems promising [7,11,26,117,144], both in the development of new extenders for long time liquid storage, and as an intervention for the development of thawing extenders. In this particular aspect, current extenders in use for stallion spermatozoa contain high concentrations of glucose, around 270–300 mM, these concentrations are far from being physiological, and may preclude long term preservation of liquid semen. It is well known that supraphysiological concentrations of glucose may lead to cell death [145] due to accumulation of advanced glycation end products (AGEs) [146,147,148,149]. The discovery of endocrine features in the spermatozoa also underlines the complex metabolism of these cells that represent an area of great interest for research in the coming decade [138,150]. Finally, amino-acid metabolism ought to be considered, this has been reported in fish spermatozoa, and anecdotal reports in mammals using amino-acids as semen additives support this possibility [151,152]. Additionally, indirect evidence of the role of the amino acid glutamine in stallion spermatozoa has been recently reported by our laboratory. Inhibition of the xCT antiporter, and thus increased intracellular glutamate improved sperm function in fresh extended stallion spermatozoa, but not in frozen thawed samples [83]. The amino-acid glutamine may enter the Krebs cycle and improve mitochondrial function under some circumstances [153]. Glutamine metabolism can provide considerable amounts of NADPH, through the pentose phosphate pathway, and can occur in parallel with aerobic glycolysis depending on glucose-6-phosphate availability [154]. The increase in sperm functionality after using the xCT antiporter inhibitor sulfasalazine can be explained through this mechanism. 

## 7. Consequences of Redox Deregulation 

**Figure 1:**
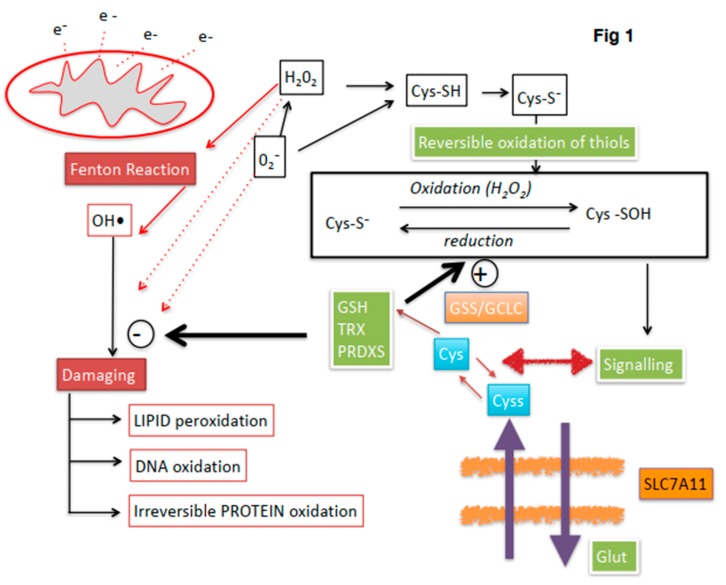
Overview of redox regulation in stallion spermatozoa. Electron (e^−^) leakage at the mitochondria is one of the main sources of ROS. Mechanisms to maintain redox homeostasis include thioredoxin (TRX) and peroxiredoxin (PRDX) systems and gluthatione (GSH) (green boxes). The stallion spermatozoa can incorporate cystine (cyss) (blue boxes), through the SlC7A11 x-CT antiporter by exchange for intracellular glutamate (Glut). Cystine is reduced in the cytoplasm to Cysteine and contribute to the intracellular GSH pool by the action of the enzymes involved in the synthesis of GHS, Glutathion syntethase (GSS) and glutamate cysteine ligase (GCLC); this mechanism has been described only in horses. Controlled levels of ROS regulate sperm functionality through reversible oxidation of thiols in cysteine containing proteins (blank boxes). If redox regulation is lost, irreversible oxidation of thiols and oxidative attack to lipids DNA and proteins occurs leading to sperm malfunction and finally death (red boxes). The hydroxyl radical (OH•) is the most damaging ROS, produced by the Habor–Weiss/Fenton reaction.

In accordance with the current biochemical literature, redox regulation is tightly regulated in the spermatozoa, with interactions between spermatic metabolism, mitochondrial production and scavenging of ROS. A summary of current knowledge on redox regulation in stallion spermatozoa is presented in Figure 1. Many factors can deregulate this complex network in humans and other animals, including aging, exposure to toxins, particularly alcohol and tobacco in humans, poor diet, lack of physical activity and systemic diseases including obesity and diabetes [30,155,156,157,158]. Also, current sperm biotechnologies such as cryopreservation cause redox deregulation of spermatozoa, mainly through a severe mitochondrial osmotic stress [110,118,128,159,160]. Deregulation of redox homeostasis has a profound impact on sperm physiology and fertility, all spermatic compartments and functions may be affected. Moreover, impacts on the embryo and the offspring may also occur. 

## 8. Effects on Lipids

**Figure 2:**
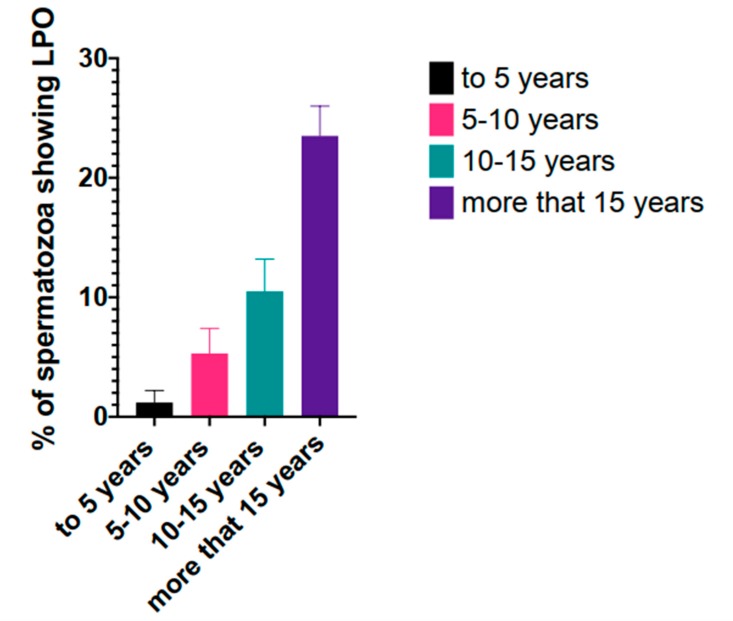
Effect of stallion age in the peroxidation of sperm membranes, semen was collected from stallions of different ages (to 5 years old, 5–10, 10–15 and more than 15 years old) and lipid peroxidation was assessed flow cytometrically after BODIPY 581/591 C11, as seen in the figure, lipid peroxidation increases with age.

Lipid peroxidation is well recognized as a consequence of redox deregulation and loss of redox homeostasis in spermatozoa. In the stallion model, lipid peroxidation occurs as a consequence of aging (Figure 2) and sperm biotechnologies such as cryopreservation and chromosomal sex sorting [89,109,110,161,162,163,164]. Deregulation of redox signalling, aging and cell senescence is well documented, and aged stallions show increased peroxidation of the lipids in the sperm membranes. Cryopreservation leads to a paradoxical situation, while osmotic induced damage in the mitochondria may lead to reduced production of ROS, lipid peroxidation increases after freezing and thawing. On the other hand, spermatozoa that withstands cryopreservation better is also characterized by increased production of ROS [31]. Lipid peroxidation (LPO) occurs after the oxidative attack of lipids, mainly the phospholipids and cholesterol of the membranes. Interestingly, LPO induces changes in the permeability and fluidity of the membranes that can be easily monitored using probes like YoPro-1 [165,166]. LPO results in the production of lipid hydroperoxides, which are unstable and decompose to more stable and less reactive secondary compounds [167,168,169]. Lipid peroxidation occurs in three phases, in the *initiation* phase abstraction of H• from a lipid chain (LH) gives a lipid radical (L•). Formation of L• is favored in the membrane of the horse spermatozoa due to their abundance in PUFAs [170,171], in this type of lipid the resulting radical is resonance stabilized [167]. Following *initiation* the *propagation* phase continues and the lipid radical reacts with oxygen to generate a lipoperoxyl radical (LOO•), that reacts with a lipid to yield a L• and a lipid hydroperoxyde (LOOH), these are unstable molecules that generate new peroxyl and alkoxyl radicals and decompose to form secondary products [168]. Finally the reaction ends when it gives a non-radical, or non-propagating species [169]. Among the secondary products formed upon lipid peroxidation of the polyunsaturated fatty acids (PUFAs) of the sperm membranes, aldehydes have received special attention due to their toxicity to spermatozoa [109,110,172,173,174,175,176,177,178,179]. Depending on the oxidation of different PUFAs, distinct compounds can originate, malondialdehyde originates from the oxidation of PUFAs containing at least three double bonds, like arachidonic acid. 4 hydroxy-2(E)-nonenal (4-HNE) originates from the oxidation of ω6 fatty acids. The composition of the sperm membrane, suggests that 4-HNE should be the prevalent compound upon LPO, since docosopentanoic acid (C22: 5ω6) is the predominant PUFA in the phospholipids of stallion spermatozoa [170]. Interestingly, recently, seasonal variation in the lipid composition of the sperm membranes has been reported [180]. It should also be noted that 4-HNE, while triggered by an initial oxidative step, can later continue independent of oxidative stress and continues providing a source of ω-6 fatty acids is available [181]. 4-hydroxynonenal reacts with GSH by Michael addition to form GSH conjugates, and although this reaction can happen spontaneously it occurs much faster in the presence of glutathione-*S*-transferases. Also, the aldehyde function of 4-HNE can be reduced into alcohol or oxidized into acid, with the participation of alcohol dehydrogenase and aldehyde dehydrogenase, forming 1,4-dihydroxynonene and 4-hydroxynonenoic acid, which can undergo beta oxidation [167]. The role of GSH and aldehyde dehydrogenase has recently been investigated in stallion spermatozoa in relation to oxidative stress [107,109,110,175], suggesting that these mechanisms for 4-HNE detoxification are of pivotal importance for spermatic function. The relation between GSH and 4-HNE in cryopreserved stallion spermatozoa suggest that GSH is effectively a major mechanism for detoxifying 4-HNE [110]. Also, aldehyde dehydrogenase has proven to be a major detoxifying mechanism for 4-HNE in stallion spermatozoa [175]. Lipid peroxidation has been traditionally detected using BODIPY dyes [89,182]; however, its dual fluorescence and its lipid binding can make this dye difficult to interpret upon flow cytometry analysis. More recently, lipid peroxidation is being detected using antibodies against 4-hydroxynonenal (4-HNE) [110,175,183]. The availability of secondary antibodies marked with different probes makes this technique suitable for multicolor panels, and to study the relation between increased levels of 4-HNE and sperm functionality using multiparametric analysis. Mass spectrometry is also a suitable tool for the study of lipid peroxidation induced changes in the spermatozoa and has recently been used in our laboratory to monitor GSH [107]. 

## 9. Effects on Proteins

Oxidative modifications of structural and functional proteins are one of the major factors involved in protein dysfunction. Protein carbonyl content is a commonly used biomarker of oxidative damage of proteins. Toxic adducts derived from LPO can diffuse through membranes allowing the reactive aldehydes to covalently modify proteins [173,174,184,185]. In addition to advanced lipid peroxidation end products (ALEs), products derived from the glycoxidation of carbohydrates, that will form advanced glycation end products (AGEs) can also induce protein carbonylation [169]. There is an excellent recent review of this particular topic focused on the spermatozoa [51] and the reader is referred to it for complete details.

## 10. Oxidative DNA Damage

Spermatozoa harbor the haploid paternal genome and also important epigenetic information with regulatory roles for early embryo development [186]. Recently, it has been reported that biotechnologies such as cryopreservation damage sperm genes with important roles in fertilization and early embryo development, even in the absence of detectable DNA fragmentation [187,188]. Cryopreservation can also damage the sperm epigenome [189]. Many assays have been developed to investigate DNA integrity in the spermatozoa [190,191]. It is considered that most of the DNA damage is caused by an oxidative mechanism. Oxidation of nucleotides can cause abasic pairs in DNA, increasing the risk of replication errors. Loss of a base in DNA, i.e., creation of an abasic site leaving a deoxyribose residue in the strand, is a frequent lesion that may occur spontaneously, or under the action of radiation or alkylating agents, or enzymatically as an intermediate in the repair of modified or abnormal bases. The abasic site lesion is mutagenic or lethal if not repaired. From a chemical view point, the abasic site is an alkali-labile residue that leads to strand breakage through beta- and delta- elimination [192,193]. More recently, multiple consequences of the electrophilic nature of abasic lesions have been revealed [194], and oxidized abasic sites are nowadays considered irreparable, leading to the most deleterious form of DNA damage, inter-strand cross links and double strand breaks [195,196]. Detection of oxidized nucleotides in sperm with flow cytometry has been reported using a specific antibody against the oxidative derivative of guanosine, 8-hydroxyguanosine [109,197], and threshold values for fertility have recently been reported in humans [198]. Another newly developed flow-cytometry-based assay, for evaluation of oxidative stress in sperm DNA, is the γHA2AX assay [199]. Although most histones are replaced by protamines, a small fraction remain in the nucleosome (5-15% in humans). This fraction contains the H2AH histone that is phosphorylated in Ser139 when under oxidative stress. The detection of γHA2AX (the phosphorylated form of the histone) has proven to be more sensitive than the TUNEL assay to detect DNA fragmentation, and also to be better correlated with pregnancy outcome in humans [200].

## 11. Impact of Early Embryo Development (EED)

Fecundation of the egg by spermatozoa with compromised redox regulation or experiencing non-lethal oxidative stress has important consequences with regard to embryo viability and the health and well-being of the offspring [201]. Assisted reproductive technologies such as in vitro fertilization and ICSI are associated with an increased incidence of birth defects in offspring [202]. Animal studies indicate that fecundation with spermatozoa experiencing oxidative stress may cause embryonic death [203], an effect that has been linked to oxidative damage in the spermatozoa [204]. Recent research from our laboratory has compared the effect of cryopreservation on the transcriptome of early equine embryos [205]. Using the same ejaculate, half processed as fresh sperm and the other half frozen and thawed, we obtained embryos from the same mare and stallion after artificial insemination with the aliquot of fresh sperm and, in the mare’s next cycle using the frozen thawed semen aliquot. The transcriptional profile of embryos obtained with frozen thawed spermatozoa differed significantly from that of embryos obtained with the fresh sperm aliquot of the same ejaculate. Significant downregulation of genes involved in biological pathways related to the gene ontology (GO) terms *oxidative phosphorylation, DNA binding, DNA replication,* and *immune response*. Interestingly, many genes with reduced expression were orthologs of genes in which knockouts are embryonic lethal in mice [205]. While the exact mechanism behind these changes remains to be elucidated, redox deregulation and oxidative stress in the spermatozoa seem to be an important factor. The spermatozoa is known to carry proteins [201], and numerous ncRNAs [206] to the oocyte, with important functions in early embryogenesis. However, it has recently been reported that caput epidydimal mouse sperm, which has not yet incorporated RNAs, can support full development [207]. The impact of redox deregulation on sperm proteins is well recognized and has recently been reviewed [51,208], so it is not unlikely that oxidized proteins can be incorporated by the embryo impacting its development. Recently, preimplantation proteins in the human embryo with potential sperm origin have been identified [201]. In particular, 93 different proteins have been proposed as related to zygote and early embryo development before implantation in humans, moreover up to 560 sperm proteins with known roles in the regulation of gene expression in other cells or tissues have also been identified [201]. Even though further investigation is needed in this field, oxidative damage to sperm proteins with important functions during early embryo development may occur. Further supporting this hypothesis is the fact that biological processes such as *DNA binding and replication,* and *Histone Acetylation* were downregulated in embryos obtained with cryopreserved spermatozoa [205], and many of the proteins mentioned above have roles in these processes [201].

## 12. Concluding Remarks

Redox regulation plays a major role in controlling sperm functionality, recent research is unveiling the existence of sophisticated redox regulation systems that may constitute targets for the treatment of the male factor subfertility. In addition, the interaction between metabolism and redox regulation may offer alternatives to traditional methods of sperm conservation. The increasing use of proteomic techniques in research in spermatology will provide significant advances in the understanding of redox regulation in the spermatozoa in coming years.
